# Surgical treatment of liver echinococcosis

**DOI:** 10.25122/jml-2022-0268

**Published:** 2022-11

**Authors:** Farkhod Radjabovich Yakubov, Duschan Shukhratovich Sapaev

**Affiliations:** 1 Department of General Surgery, Urgench Branch of Tashkent Medical Academy, Urgench, Republic of Uzbekistan

**Keywords:** laparoscopic echinococcectomy, PAIR, PEVAC

## Abstract

This article is based on the treatment results of primary and recurrent epidural infection among 1230 patients treated at three medical institutions: Khorezm Regional Multidisciplinary Medical Center, Clinic of Andijan State Medical Institute, Republican Specialized Scientific and Practical Medical Center for Surgery, between 2015–2020. The comparison group included 621 patients (from 2015 to 2017) who underwent a retrospective analysis. In comparison, the main group of the study included 609 patients (from 2018 to 2020). In the main group of patients, traditional echinococcectomy (EE) was performed in 80.1% of cases, LapEE in 12.3%, and PAIR and PEVAC techniques in 7.6%. The overall incidence of complications from the residual cavity in the comparison group was 36.4% after the PAIR technique, 39.1% after the PEVAC technique, 21.7% after LapEE, and 6.9% (37 out of 503) after traditional and resection EE. The proposed algorithm for selecting a method for treating exocrine pancreatic insufficiency (EPI) made it feasible to optimize tactical approaches to perform traditional and minimally invasive interventions, which ensured a decrease in the incidence of complications from the residual cavity and, accordingly, the need for repeated minimally invasive and open interventions and conservative therapy.

## INTRODUCTION

According to the World Health Organization (WHO), vector-borne and parasitic diseases affect more than 2 billion people worldwide. For example, liver echinococcosis (LE), a cestode zoonosis, the etiological factor of which is tapeworms of the genus Echinococcus, affects up to 200 people per 100 thousand of the population annually, causing a cystic form of the disease [1]. The main characteristic of LE is its high invasion in cases of ruptured cystic formation and the spread of helminth eggs throughout the human body [2].

As a result of various mechanisms, complications of the disease develop in 20–40% of cases, including damage to the bile ducts (up to 42%), compression of the hepatic, portal or vena cava, cyst rupture (1–8%), bacterial superinfection (7%), severe anaphylactic phenomena (1%), cystobronchial fistulas [3].

To date, numerous studies have indicated the high effectiveness of antiparasitic drugs in the conservative treatment of LE. However, in surgical treatment, there is still no single generally accepted protocol that considers the form and morphological stage of the disease, which is associated with an insufficient evidence base in terms of developing indications for various surgical approaches (minimally invasive, puncture, drainage procedures, traditional operations) and methods for eliminating and treating the residual cavity (RC) after echinococcectomy (EE). The aim of this study was to scrutinize in a comparative aspect the summary results of surgical treatment of LE.

## MATERIAL AND METHODS

The study was performed as part of an open multicenter retrospective analysis for the comparison group and prospective for the main group with randomization by location, size, and stage of development of echinococcal liver disease, as well as by the type of minimal invasive or open traditional surgical treatment. The work is based on the results of treatment of primary and recurrent LE in 1230 patients treated between 2015–2020 in three medical institutions with different regional localization in the Republic of Uzbekistan:

- Khorezm Regional Multidisciplinary Medical Center: 365 (29.7%) patients;
- Clinic of Andijan State Medical Institute: 352 (28.6%) patients;
- State Institution Republican Specialized Scientific and Practical Medical Center for Surgery named after Academician V. Vakhidov: 513 (41.7%) patients.

The criteria for inclusion in the study were: patients aged >18; the presence of a parasitic lesion only within the liver; primary and recurrent forms of LE, solitary and multiple cysts; the size of echinococcal cysts ≥4 cm, including cases with cysts up to 5 cm in case of ineffectiveness of 2–3 courses of chemotherapy; stages of cyst development corresponding to type I-II-III CE (according to WHO 2010) [1], as well as type IV CE cysts in a complicated course (abscessing clinic) and consent of patients to minimally invasive or traditional surgical treatment of LE.

Criteria for exclusion in the study were: patients aged <18; LE defined as a simultaneous diagnosis with the need to treat the underlying disease; a combination of LE with cysts of extrahepatic localization (other organs of the abdominal cavity); echinococcal cysts up to 5 cm, not previously treated conservatively (therapeutic chemotherapy was recommended), and stages of cyst development, corresponding to CE IV or V type (according to WHO, 2010) [1] without signs of a complicated course (monitoring in dynamics was recommended).

All patients were stratified into two groups. The comparison group included 621 patients treated from 2015 to 2017, for whom we performed a retrospective analysis of the results of various interventions for LE. Based on the data obtained, the tactical approaches for choosing minimally invasive or traditional surgical treatment of LE were optimized, and the clinical efficacy was evaluated in the main study group – 609 patients treated between 2018 and 2020. The main principles for advancing the tactical approaches regarding the surgical treatment of LE in the main group were:

- Specifying the indications for isolated or combined with other methods of performing puncture (PAIR) and puncture-draining (PEVAC) methods of treating LE, depending on the location, size, and stage of development of the hydatid lesion;
- Optimizing laparoscopic EE (LapEE) according to the same criteria and the technical approaches for the treatment of residual cavity (RC) and the volume of pericystectomy to create adequate abdominalization and reduce the incidence of complications from the RC;
- Active use in traditional EE of complete or partial elimination or wide abdominalization of RC with a decrease in the proportion of drainage to reduce the incidence of complications from RC;
- Expanding the indications for the combined treatment of multiple LE, in the presence of deep intraparenchymal cysts, by combining surgical and minimally invasive (PAIR and PEVAC) methods;
- Improving surgical LE treatment by using the domestic drug “FarGALS”.

Most patients in the comparison group (64.6%; 401 out of 621) and the main group (57.8%; 352 out of 609) were diagnosed with primary solitary LE. According to the frequency of occurrence, primary-multiple, recurrent solitary, and recurrent-multiple forms of LE were identified. There were more female patients in the comparison group (61.4%; 381 out of 621) and the main study group (63.9%; 389 out of 609). In terms of age, the majority of patients in the comparison group (75.5%; 469 out of 621) and in the main group (74.2%; 452 out of 609) were of working age, from 18 to 44 years.

Echinococcosis in the form of a primary solitary cyst was detected in the right lobe of the liver in most cases, both in the comparison group (59.6%; 320 cases) and in the main group (51.6%; 314 cases).

In the case of a recurrent solitary cyst, pathology was regularly detected in the right lobe of the liver: in 16.6% (101) and 12.9% (80) of cases in the comparison group and the main group, respectively. Similar statistics were noted in cases of primary multiple and recurrent meningeal echinococcosis. In total, the incidence of echinococcosis in the right lobe of the liver was 83.7% in the comparison group and 79.1% in the main study group.

We also observed complicated forms of LE: 52 (8.4%) patients in the comparison group and 65 (10.7%) patients in the main group. Among the complicated forms of echinococcosis of the liver, cases of cyst suppuration prevailed, observed in 37 (6.0%) patients in the comparison group and 46 (7.6%) in the main group.

In solitary LE, both in the comparison group (38.8%; 189 of 487) and in the main group (38.0%; 174 of 458), cysts 8–10 cm in size were most often detected. The second most frequent were cysts larger than 10 cm: in 29.2% of cases in the comparison group and 33.2% in the main group.

With multiple LE in the comparison group, cysts 6–8 cm in size were frequently identified (40.0%; 189 out of 473), while in the main group, cysts 8–10 cm in size were more often diagnosed (34.6%; 179 out of 517), and sizes 6–8 cm accounted for 32.1% (166 out of 517).

Patients were also classified according to the sonographic classification recommended by WHO (2010) [1]. Intermediate stages of the disease (CE III) accounted for half of the cases, both in the comparison group (51.4%; 319 out of 621) and in the main group (54.4%; 331 out of 609).

The inactive stage of the disease (CE IV) was the least detected: in 5.3% (33 of 621) cases in the comparison group and 6.4% (39 of 609) in the main group.

The computer program STATISTICA (StatSoft.Inc) was used for the statistical processing of the study material. Sample size estimation, power analysis, and statistical error analysis were carried out using parametric and non-parametric analysis. Normally distributed quantitative variables were assayed using parametric tests (Student's t-test and repeated measures analysis of variance). Comparison of nominal data was carried out using Pearson's χ2 test. In the case of the analysis of four-field tables with the expected phenomenon in at least one cell less than 10, the χ2 criterion with the Yates correction was calculated. In cases where the number of expected observations in any of the cells of the four-field table was less than 5, Fisher's exact test was used to assess the significance level of disparities. The value of Fisher's exact test p>0.05 indicated the absence of statistically significant differences.

## RESULTS

**Figure 1:**
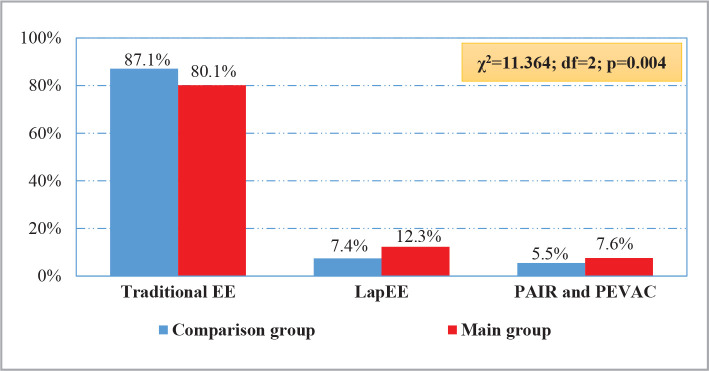
Distribution of patients according to the main type of LE surgical treatment.

According to the main type of LE surgical treatment (Figure 1), the proportion of minimally invasive interventions was greater in the main group than in the comparison group: traditional EE was performed in 80.1% (488 of 609) cases, LapEE in 12.3% (75 of 609), and PEVAC in 7.6% (46 out of 609). However, in the comparison group, LapEE was performed in 7.4% (46 out of 621) of cases, and PAIR and PEVAC in 5.5% of patients (34 of 621) (χ2=11.364; df=2; p=0.004).

In the main group of patients, EE with complete or partial elimination of RC was performed with a greater frequency (62.1% *vs*. 41.7% in the comparison group), both in solitary LE (67.0% *vs*. 42.5% in the comparison group), and with multiple LE (47% *vs*. 38.8% in the comparison group). Accordingly, the proportion of EE with drainage and/or abdominal RC was lower (27.9%) in the main group (24.5 with solitary LE and 38.4% with multiple) than in the comparison group (50.9%) (50.7% in solitary LE and 51.5% in multiple EP).

In contrast, the frequency of using puncture or drainage methods for treating RC increased from 5.5% (34 out of 621) in the comparison group to 7.6% (46 out of 609) in the main group of patients.

In total, in the main group with multiple LE, 517 cysts were operated, of which in most cases (33.1%; 171 out of 517) complete suturing of the RC was performed, while in the comparison group of 473 operated cysts, this method was used only in 14.4% (68 of 473) of cases, and in most (39.5%; 187 of 473) cases, drainage of the RC was performed.

Additionally, the frequency of performing PAIR increased from 4.0% (19 out of 473) in the comparison group to 7.9% (41 out of 571) in the main group and PEVAC from 3.4% (16 out of 473) in the comparison group to 7.0% (36 out of 571).

The overall incidence of complications from RC in the comparison group was 36.4% (4 out of 11) after the PAIR technique, 39.1% (9 of 23) after PEVAC, 21.7% (10 of 46) after LapEE, and 6.9% (37 out of 503) after conventional and resection EE.

The overall incidence of complications from RC was significantly lower in the main group: 15.4% (2 out of 11) after the use of the PAIR, 12.1% (4 out of 29) after PEVAC (χ2=5.547; df=1; p=0.019), 8.0% (6 of 69) after LapEE (χ2=4.690; df=1; p=0.031) and 3.1% (15 of 473) after conventional and resection of EE (χ2=7.619; df=1; p=0.006).

**Figure 2:**
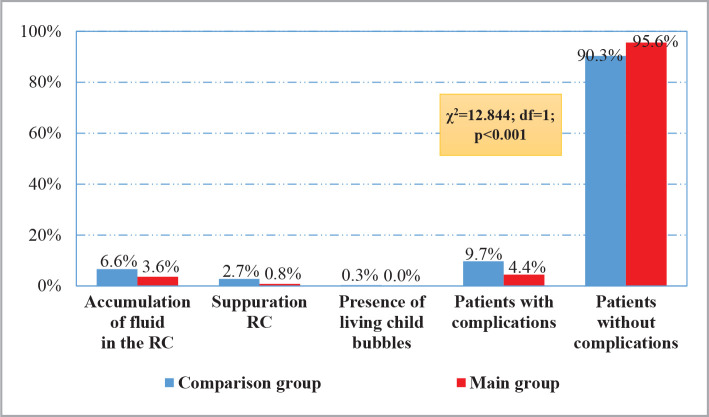
Summary frequency of complications from RC.

The summary data on the incidence of postoperative complications from RC (Figure 2) showed fluid accumulation in the RC in 3.6% of cases (22 out of 609) in the main group and 6.6% (41 out of 620) in the comparison group. Suppuration of the RC occurred in 0.8% (5 out of 609) of cases in the main group and 2.7% (17 out of 620) in the comparison group. Another complication was the presence of living child bladders, which occurred in 0.3% (2 of 620) in the comparison group and was not detected in the main study group. A complicated postoperative course was noted in 4.4% (27 of 609) cases in the main group, which was significantly lower (χ2=12.844; df=1; p<0.001) than in the comparison group (9.7%).

In the long term, after various types of operations for LE, 488 cases were followed up in the comparison group and 518 cases in the main group, of which traditional EE was performed in 82.4% of cases in the comparison group and 74.9% in the main group of patients. After LapEE, the long-term period was monitored in 9.0% (11 out of 488) of patients in the comparison group and 13.9% (72 out of 518) in the main group (Table 1).

**Table 1:** Distribution of patients followed up in the long term.

Intervention	Comparison group		Main group	
	n	%	n	%
Traditional EE	402	82.4%	388	74.9%
Ideal EE	11	2.3%	13	2.5%
LapEE	44	9.0%	72	13.9%
PAIR	9	1.8%	13	2.5%
PEVAC	22	4.5%	32	6.2%
Total	488	100.0%	518	100.0%

The surgical treatment results of LE in the comparison group were followed up for more than 3 years in 34.0% (166 out of 488) cases and 6.4% in the main group.

Disease recurrence (Table 2) was noted within a year after surgery in 8.6% (7 out of 488) cases in the comparison group and 1.5% (2 out of 518) in the main group. In terms of 1 to 2 years, the frequency of diagnosed recurrence of LE was 9.2% (10 out of 488) and 4.1% (8 out of 499) in the comparison group and the main group, respectively. 3 or more years after the operation, recurrence of LE was noted with a frequency of 5.4% in the comparison group and 3.0% in the main group. The overall relapse rate in the main group was 3.7% (19 out of 518), which was significantly lower (χ2=7.323; df=1; p=0.007) than in the control group (7.6%; 37 out of 488).

**Table 2:** Frequency of diagnosed disease recurrence.

Time of observation	Comparison group		Main group	
	n	%	n	%
Up to a year	7	8.6%	2	1.5%
1–2 years	10	9.2%	8	4.1%
2–3 years	11	8.3%	8	5.1%
3 and more years	9	5.4%	1	3.0%
Total	37	7.6%	19	3.7%
No relapse	451	92.4%	499	96.3%
χ2 between groups	7.323; df=1; p=0.007			

Depending on the type of surgical treatment for LE in the comparison group, disease recurrence (Table 3) was noted with almost equal frequency both with traditional and minimally invasive interventions, while in the main group, most cases of LE recurrence were noted after ideal EE (7.7%) and PAIR methods (7.7%).

**Table 3:** Distribution of LE relapses depending on the type of treatment.

Intervention	Comparison group		Main group	
	n	%	n	%
Traditional EE	29	7.2%	14	3.6%
Ideal EE	1	9.1%	1	7.7%
LapEE	4	9.1%	2	2.8%
PAIR	1	11.1%	1	7.7%
PEVAC	2	9.1%	1	3.1%
Total relapses	37	7.6%	19	3.7%

Furthermore, there was a statistically significant difference in traditional EE (p=0.011) between the study groups, after which the recurrence rate in the comparison group was 7.3%, while in the main group it was 3.7%. The recurrence rate was lower after other interventions in the main group but without statistical significance (Table 4).

**Table 4:** Frequency of a LE recurrence and relapse-free course depending on the type of treatment.

Intervention	Comparison group				Main group				χ2	p
	Relapse		Without relapse		Relapse		Without relapse			
	n	%	n	%	n	%	n	%		
Traditional EE	30	7.3%	383	92.7%	15	3.7%	386	96.3%	6.493	0.011
LapEE	4	9.1%	40	90.9%	2	2.8%	70	97.2%	2.219	0.137
PAIR & PEVAC	3	9.7%	28	90.3%	2	4.4%	43	95.6%	0.818	0.366
Total relapses	37	7.6%	451	92.4%	19	3.7%	499	96.3%	7.323	0.007

In the comparison group, the localization of recurrent echinococcosis with a higher frequency (4.3%) was noted in the same zone, and in the main group, relapse (1.7%) was most often observed in other segments within the same lobe of the liver (Table 5).

**Table 5:** Localization of echinococcosis recurrence relative to the primary location.

Recurrence localization	Comparison group		Main group	
	n	%	n	%
In the same zone	21	4.3%	7	1.4%
In other segments within the share	10	2.0%	9	1.7%
In another share	2	0.4%	1	0.2%
In the liver and the abdomen	4	0.8%	2	0.4%
No relapse	451	92.4%	499	96.3%
χ2 between groups	9.592; df=4; p=0.048			

Table 6 demonstrates that after operations for primary LE in the comparison group, the recurrence rate was 6.4% (25 out of 367), while after interventions for recurrent LE, the recurrence rate was twice as high and amounted to 12.5% (12 out of 84). In the main study group, these figures were lower and amounted to 2.7% (11 out of 391) and 6.9% (8 out of 108) in primary and recurrent LE, respectively. It is noteworthy that the most frequent recurrence occurred in the same area of the operation among patients from the comparison group and in other segments within the share – among patients from the main study group.

**Table 6:** The frequency of recurrence of LE depending on the primary form (primary or recurrent LE).

Intervention	Primary LE			Recurrent LE		
	n	Relapse	%	n	Relapse	%
**Comparison group**						
In the same zone	392	13	3.3%	96	8	8.3%
In other segments within the share		8	2.0%		2	2.1%
In another share		1	0.3%		1	1.0%
In the liver and the abdomen		3	0.8%		1	1.0%
**Total relapses**		**25**	6.4%		**12**	12.5%
**Without relapse**		**367**	93.6%		**84**	87.5%
χ2	4.125; df=1; p=0.043					
**Main group**						
In the same zone	402	3	0.7%	116	4	3.4%
In other segments within the share		6	1.5%		3	2.6%
In another share		1	0.2%		0	0.0%
In the liver and the abdomen		1	0.2%		1	0.9%
**Total relapses**		**11**	2.7%		**8**	6.9%
**Without relapse**		**391**	97.3%		**108**	93.1%
χ2	4.410; df=1; p=0.036					
χ2 between groups	6.079; df=1; p=0.014			1.930; df=1; p=0.165		

Among all relapses in the comparison group (37 cases) in the same zone, relapse occurred in 56.8% of cases, while in the main group (19 cases) – 36.8% of cases, where the majority were relapses in other segments within the share (47.4%). In another lobe of the liver (5.4% and 5.3%), as well as in the liver and the abdominal cavity (10.8% and 10.5%), relapses occurred with almost equal frequency, both in the comparison group and in the main study group. At the same time, there were no relapses in another lobe of the liver after operations for recurrent LE among patients of the main group, and in the comparison group this figure was 8.3%.

However, after operations for primary LE in the main group in another lobe of the liver (9.1%), relapse occurred with a higher frequency than in the comparison group (4.0%).

## DISCUSSION

To date, the main strategies for the surgical treatment of LE encompass traditional and laparoscopic EE, as well as percutaneous puncture-draining interventions (PAIR and PEVAC techniques). The purpose of surgical treatment is direct inactivation of the parasite, evacuation of the cyst along with the removal of the germinal layer, pericystic zone, prevention of the peritoneal spread of scolex, and treatment of RC, thereby solving the problems of complications and the contingency of recurrence [4]. However, with all treatments, recurrence rates range from 7% to 25%, morbidity rates range from 12% to 84%, and mortality rates range from 0.5 to 6.5% [5].

Recent meta-analyses indicate that radical surgery is preferred to reduce the risk of postoperative abdominal infection, biliary fistulas, and overall mortality [6].

Also, in this study, traditional EE was performed in 80.1% (488 of 609) cases from the main group and LapEE in 12.3% (75 of 609).

According to a retrospective study by J.M. Ramia Angel et al. (2020), there was no significant difference in postoperative complications between different radical approaches in LE surgery [7].

Alternatively, according to the data in the main group, the overall complication rate was 8.0% (6 out of 69) after LapEE and 3.1% (15 out of 473) after conventional and resection EE.

In cases of severe biliary tract lesions, vessels with lobar atrophy or recurrence, liver resection is used, which ensures the removal of any smaller satellite lesions. However, indications for liver resection in LE have not found a consensus. In a study by W. Patkowski et al. (2017), after resection interventions (bisegmentectomy, right-sided and left-sided hemihepatectomy) in LE, the complication rate (biliary fistula and subdiaphragmatic abscess) was 3.4%. No lethal outcomes were noted, and survival after 1, 5, and 10 years were 100%, 90.9%, and 87.9%, respectively [8].

Recently, puncture-draining methods for treating epilepsy, such as PAIR and PEVAC, have become prevalent. Hence, the results of a prospective randomized study by O. Akhan et al. (2020), which compared the long-term results of puncture-drainage methods for the treatment of epilepsy, revealed that for the treatment of echinococcal cysts of the liver CE1 and CE3, preference should be given to the PAIR method [9].

## CONCLUSION

Improving the tactical and technical aspects of LE surgical treatment made it possible to decrease the incidence of complications from RC from 9.7% to 4.4% (χ2=12.844; df=1; p<0.001). Accordingly, the need for repeated minimally invasive interventions decreased from 2.9% to 1.0%, open surgery from 1.3% to 0.2%, and conservative therapy from 5.5% to 3.3% (χ2=15.401; df=3; p=0.002) was accomplished.

Dynamic follow-up three years after surgery demonstrated that optimizing the tactical approaches for choosing EE decreased the risk of recurrence after traditional operations from 7.3% to 3.7%, laparoscopic interventions – from 9. 1% to 2.8%, puncture-drainage techniques – from 9.7% to 4.4%, in general, providing a decrease in this indicator from 7.6% to 3.7% (χ2=7.323; df=1; p=0.007).
